# The Role of Na+/Ca2+ Exchanger 1 in Maintaining Ductus Arteriosus Patency

**DOI:** 10.1038/s41598-017-10377-z

**Published:** 2017-08-29

**Authors:** Minghui Li, Chuan Jiang, Lincai Ye, Shoubao Wang, Haibo Zhang, Jinfen Liu, Haifa Hong

**Affiliations:** 1 Department of Thoracic and Cardiovascular Surgery, Shanghai Children’s Medical Center, Shanghai Jiaotong University School of Medicine, Shanghai, 200127 China; 2 Institute of Pediatric Translational Medicine, Shanghai Children’s Medical Center, Shanghai Jiaotong University School of Medicine, Shanghai, 200127 China; 3 Shanghai Pediatric Congenital Heart Disease Institute, Shanghai Children’s Medical Center, Shanghai Jiaotong University School of Medicine, Shanghai, 200127 China

## Abstract

Patency of the ductus arteriosus (DA) is crucial for both fetal circulation and patients with DA-dependent congenital heart diseases (CHD). The Na^+^/Ca^2+^ exchanger 1 (NCX1) protein has been shown to play a key role in the regulation of vascular tone and is elevated in DA-dependent CHD. This current study was conducted to investigate the mechanisms underpinning the role of NCX1 in DA patency. Our data showed NCX1 expression was up-regulated in the DA of fetal mice. Up-regulation of NCX1 expression resulted in a concomitant decrease in cytosolic Ca^2+^ levels in human DA smooth muscle cells (DASMCs) and an inhibition of the proliferation and migration capacities of human DASMCs. Furthermore, treatment of DASMCs with KB-R7943, which can reduce Ca^2+^ influx, resulted in the inhibition of both cell proliferation and migration. These findings indicate that NCX1 may play a role in maintaining patent DA not only by preventing DA functional closure through reducing cytosolic Ca^2+^ level in DASMC but also by delaying the anatomical closure process. The latter delay is facilitated by the down-regulation of human DASMC proliferation and migration. It is also likely that a reduction in cytosolic Ca^2+^ levels inhibits the proliferation and migration capacities of human DASMCs *in vitro*.

## Introduction

The ductus arteriosus (DA), which connects the aorta and pulmonary artery, plays important roles in fetal circulation. This patent blood vessel shunts more than half of the deoxygenated cardiac output into the descending aorta^1^. In DA-dependent CHD infants, the patent DA(PDA) supplies oxygen to the body in left sided obstruction and provides the only way for blood to get to the lungs in the right sided obstruction, thereby increasing survival and decreases need for palliative operations^2^. A patient who depends on a PDA to survive requires an operating of some sort before discharge. Thus, maintenance of the patent status of the DA is not only crucial for fetal circulation, but is also important for infants with DA-dependent congenital heart diseases (CHD).

Currently three signaling pathways have been proposed to regulate DA patent: 1) Prostaglandin signaling; 2) ion channel pathways; and 3) cytochrome P450/endothelin-1 pathway^3^. Due to the complications of prostaglandin, such as apnea, and the uneffectiveness of cytochrome P450/endothelin-1 pathway, ion channel pathways are under extensive investigation^4^. Previously by proteomic analysis, we compared patent and constricted human DA samples, finding 132 different expression proteins, among of which 3.15% were related to ion channels^5^. The Na+/Ca2+ exchanger 1(NCX1), the dominant isoform of the NCX, is one of the different proteins. NCX1 has been shown to play a key role in regulating intracellular calcium levels in both heart and smooth muscle cells^6, 7^. Previous studies conducted by our group reported that hypoxia-induced cytosolic calcium reductions were mediated by the Na^+^/Ca^2+^ exchanger protein in fetal DA^8^. Furthermore, elevated NCX1 expression was observed in the patent postnatal DA of ductal-dependent congenital heart disease patients^9^. However, the mechanisms that underlie the NCX1-mediated regulation of the DA status are unclear.

DA closure occurs in two overlapping phases^10^: (1) functional closure of the lumen, characterized by DA smooth muscle constriction, in which cytosolic calcium concentration ([Ca2+ ]i) plays a pivotal role; and (2) anatomic occlusion of the lumen due to extensive neointimal thickening. Migration and proliferation of DASMCs are intimately involved in the second phase. Here we investigated whether NCX-1 participate DA functional and anatomic closure.

## Results

### NCX1 expression is down-regulated after birth in the mouse DA

**Figure 1:**
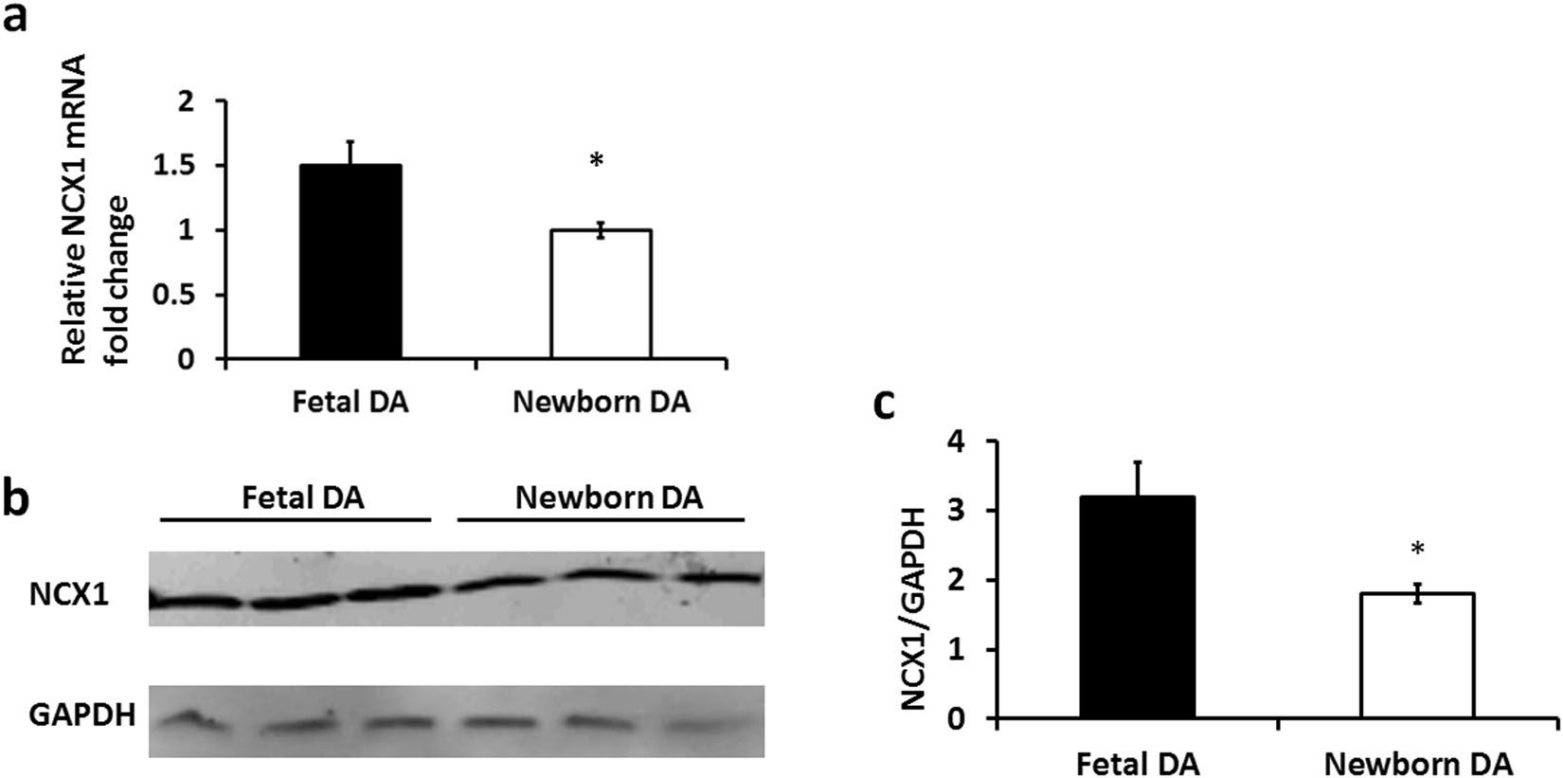
NCX1 expression in fetal mice DA is higher than in newborn mice DA. (**a**) NCX1 mRNA expression levels are apparently higher in fetal mice DA compared with newborn DA. GAPDH served as an internal control. (**b**) Cropped blots showed that NCX1 protein expression levels are significantly higher in fetal mouse DA compared with newborn DA. GAPDH served as an internal control. The error bars indicate means ± SD. *P < 0.05. (**c**) Quantitative data obtained by densitometry analysis using GAPDH as a loading control. Data presented as means ± SDs. Student’s t-tests were performed to determine statistical significance, n = 3; *p < 0.05.

20 fetal and 20 newborn mice DA (constricted, 6 hours after birth) tissue samples were collected. These tissue samples were used to compare NCX1 expression before and after birth. qPCR and Western blotting were performed to detect NCX1 mRNA and protein expression levels, respectively. Figure 1a shows that relative NCX1 mRNA levels in fetal DA are 1.5-fold greater than in newborn DA (n = 6; p = 0.03). Furthermore, NCX1 protein levels in newborn mouse DA were 41.8 percent lower than in fetal mouse DA (n = 20; p = 0.02) (Fig. 1b,c).

### Overexpression of NCX1 can reduce cytosolic Ca2+ in human DASMCs

**Figure 2:**
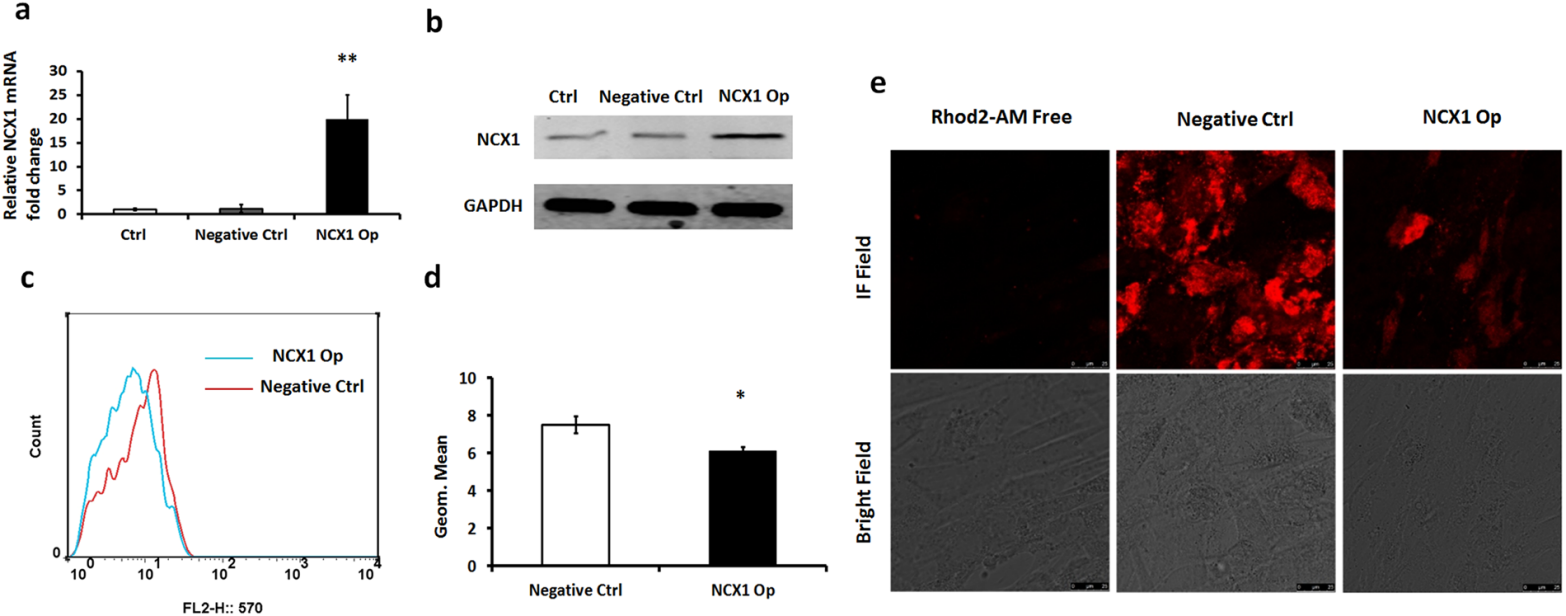
Flow cytometry assay showing apparent reduction in cytosolic calcium concentration in human DASMCs transfected with NCX1-cDNA. (**a**) Compared with the control and negative control groups, DASMCs transfected with NCX1-cDNA express higher levels of NCX1 mRNA. (**b**) Cropped blots showed that compared with the control and negative control groups, DASMCs transfected with NCX1-cDNA express higher levels of NCX1 protein. (**c**) Negative control (red) and siARHGAP26 (blue) groups are shown (n = 3). (**d**) Quantification of mean calcium fluorescence intensity showed an apparent reduction in cytosolic calcium concentration in DASMCs transfected with NCX1-cDNA. (**e**) Calcium imaging showed that after overexpression of NCX1 the fluorescence in the DASMCs which represent for the cytosolic calcium concentration was reduced compared to the negative control group. The scale bar is 25 μm. The error bars indicate means ± SD. *P < 0.05, **P < 0.01.

Cytosolic Ca^2+^ is believed to be an important secondary messenger in the regulation of vascular tone. Thus, we investigated the relationship between NCX1 and Ca^2+^. Since normally DA is closed after birth, constricted DA is considered as normal DA, in which the expression of NCX1 is relatively low. Thus we isolated human DASMCs from constricted DA tissues, which were subsequently cultured *in vitro*. We overexpressed the NCX1 gene (NCX1-Op) in human DASMCs following transfection with NCX1-cDNA. qPCR and Western blotting were subsequently utilized to determine transfection efficiency. As shown in Fig. 2a, following transfection with NCX1-cDNA, NCX1 expression was up-regulated 20-fold compared with cells transfected with control sequences (Negative Ctrl) (n = 6; p = 0.007). Western blot analyses further confirmed that NCX1 protein expression is up-regulated following transfection with NCX1-cDNA (Fig. 2b). Flow cytometry was used to detect cytosolic Ca^2+^ concentration in the associated cells (Fig. 2c). The results of this analysis revealed that the mean fluorescence intensity in NCX1-Op human DASMCs was 76.8 percent of the negative control value (n = 3; p = 0.04) (Fig. 2d). In order to confirm the results of flow cytometry, we performed calcium imaging experiment, which showed the fluorescence intensity of the DASMCs transfected with NCX1-cDNA was apparently reduced compared with negative control group (Fig. 2e). These results indicate that cytosolic Ca^2+^ is negatively correlated with NCX1 expression.

### Overexpression of NCX1 reduces human DASMC proliferation and migration

**Figure 3:**
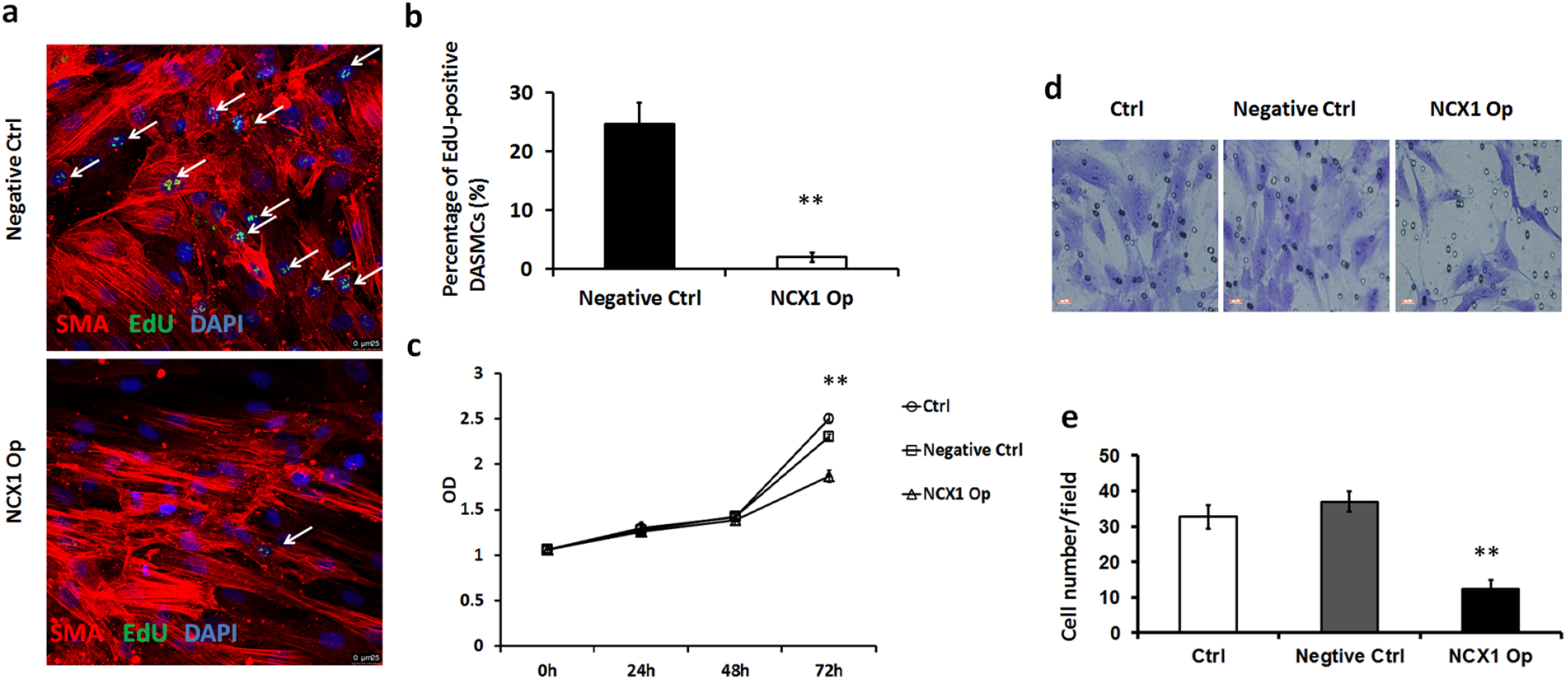
NCX1 overexpression inhibits human DASMC proliferation and migration. (**a**) Representation of EdU-positive negative control (n = 6) and NCX1-Op (n = 6) DASMCs (red, smooth muscle actin; green, EdU; blue, DAPI). The scale bar is 25 μm. (**b**) Quantification of EdU-positive DASMCs. The error bar indicates the means ± SD. **P < 0.01. n = 6. (**c**) Growth curves demonstrating that the growth of transfected DASMCs are apparently inhibited. (**d**) Giemsa staining revealed that there is a reduction in transmembrane DASMCs following up-regulation of NCX1 expression. The scale bar is 100 μm. (**e**) Numerical data for transmembrane DASMCs obtained by imageJ analysis (n = 5). Error bars indicate the means ± SD. **P < 0.01.

Cell proliferation assay (CCK-8 assay) results showed that NCX1 overexpression resulted in a decrease of 15.3 percent in the proliferation rate of human DASMCs after 72 h (n = 6; p = 0.008) (Fig. 3c). An EdU assay was performed to further validate the latter results (Fig. 3a). Following up-regulation of NCX1 at 72 h, the number of EdU-positive DASMCs was 85.9 percent lower than the negative control value (n = 6; p = 0.004) (Fig. 3b). Since SMC migration is one of the most important cellular processes in intimal cushion formation, we conducted a transwell assay to examine the role of NCX1 in human DASMC migration (Fig. 3d). As shown in Fig. 3e, upon comparison with the negative control group, overexpression of NCX1 exerted a significant inhibitory effect on the migration capacity of human DASMCs. The results of this analysis revealed that the number of transmembrane DASMCs was 32.7% of the negative control value (n = 6; p = 0.002) (Fig. 3e).

### A decline in cytosolic Ca^2+^ concentration inhibits the proliferation and migration of human DASMCs

**Figure 4:**
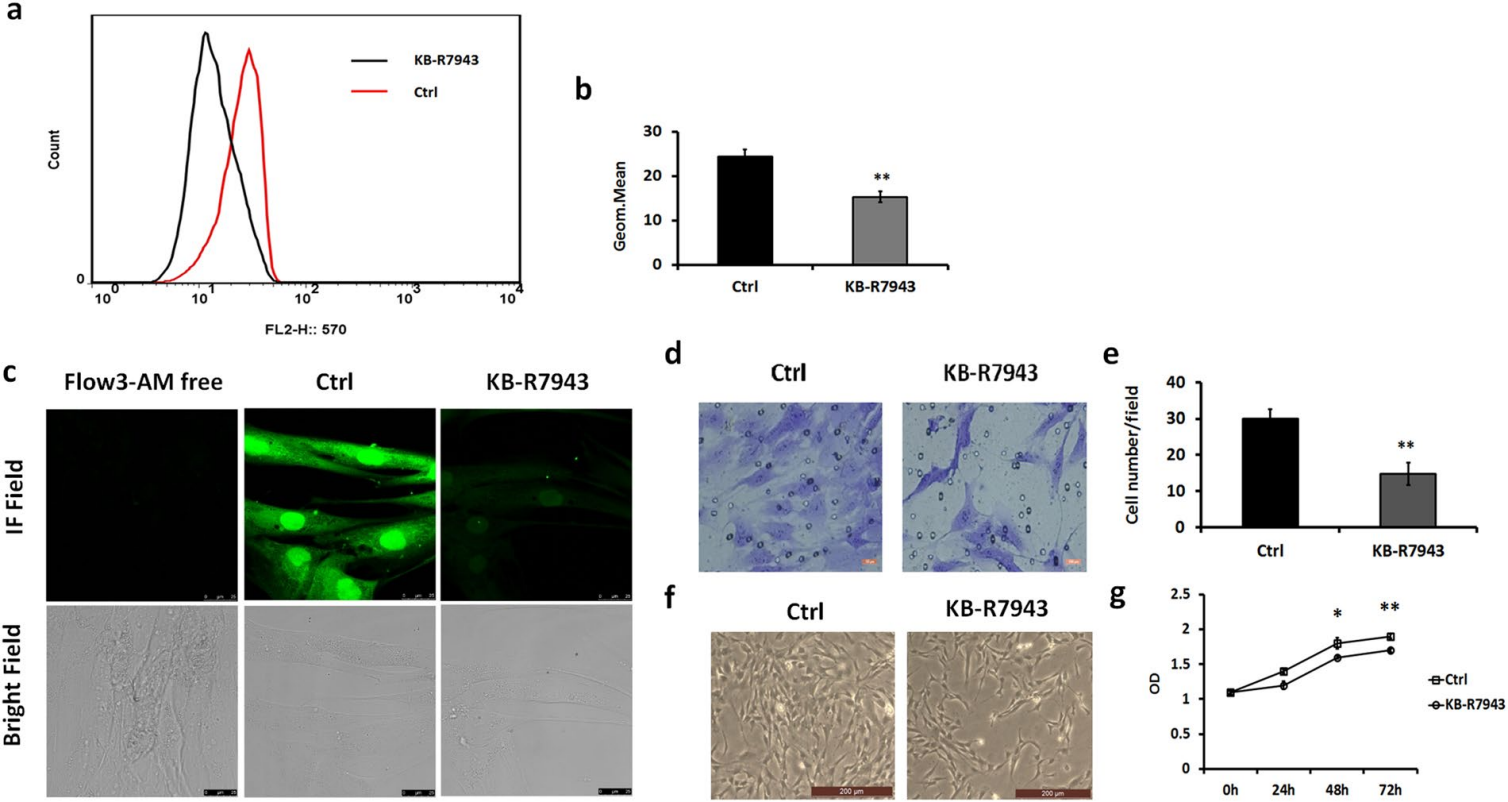
A reduction in cytosolic Ca^2+^ levels inhibits the proliferation and migration of human DASMCs. (**a**) Control (black) and test (red) groups are shown (n = 3). Normal DASMCs served as a control. DASMCs to which 10 µm KB-R7943 was added constituted the test group. (**b**) Quantification of mean calcium fluorescence intensity reveals that cytosolic calcium concentrations in DASMCs exposed to KB-R7943 are apparently reduced. (**c**) Calcium imaging showed that after added with 10 µm KB-R7943 the fluorescence in the DASMCs which represent for the cytosolic calcium concentration was reduced compared to the control group. The scale bar is 25μm. (**d**) Giemsa staining revealed a reduction in the number of transmembrane DASMCs following incubation with 10 µm KB-R7943. The scale bar is 100μm. (**e**) Numerical data for transmembrane DASMCs obtained following imageJ analysis (n = 5). (**f**) Microscopic photos demonstrating that KB-R7943-treated DASMCs grow slower than control groups. The scale bar is 200 μm. (**g**) Growth curves demonstrating a reduction in the growth of DASMCs following incubation with KB-R7943. The error bar indicate means ± SD. *P < 0.05, **P < 0.01.

In order to detect whether a reduction in cytosolic Ca^2+^ levels resulted in the concomitant inhibition of proliferation and migration capacities of human DASMCs, KB-R7943 (10 µM, consistent with our previous publication^8^, supplemental Fig. 3) was used to determine the effect of Ca^2+^. It facilitates a decrease in cytosolic Ca^2+^ mostly through inhibiting the reverse mode of NCX without affecting mitochondria respiration^11–14^. Figure 4a showed that cytosolic Ca^2+^ was reduced following the addition of 10 µM KB-R7943. The results of this analysis revealed that the mean fluorescence intensity in human DASMCs supplemented with KB-R7943 was 75% of the normal control value (n = 6; p = 0.008) (Fig. 4b). A calcium imaging assay showed that after treated with 10 µm KB-R7943 the fluorescence in the DASMCs which represent for the cytosolic calcium concentration was reduced compared with the control group (Fig. 4c). A transwell assay revealed that human DASMCs cultured *in vitro* with 10 µM KB-R7943 for 72 hours displayed inhibited migratory capacities (Fig. 4e). The results of this analysis revealed that the number of KB-R7943-supplemented transmembrane DASMCs was 53.2% of the normal control (without adding KB-R7943) value (n = 6; p = 0.001) (Fig. 4f). Figure 4g shows that the number of total KB-R7943-supplemented DASMCs (cultured for 72 h) was apparently less than for the normal control. The results of the CCK-8 assay revealed a decrease of 14.7% in the proliferation rate of DASMCs after 72 h of culture with KB-R7943 (n = 6; p = 0.002) (Fig. 4h). Hence, we hypothesize that overexpression of NCX1 inhibits DASMC proliferation and migration by decreasing cytosolic calcium levels.

## Discussion

In this study, we demonstrated for the first time that NCX1 mRNA and protein expression is apparently higher in fetal mouse DA compared with newborn mouse DA tissues. The mechanisms that underlie the NCX1-mediated regulation of human DA have not been previously studied. NCX catalyzes the bidirectional electrogenic exchange of Na^+^ for Ca^2+^ across the plasma membrane^15^. One of the exchange transport mechanisms, known as the forward mode, acts to extrude calcium in resting excitable cells. The reverse mode of NCX mediates the extrusion of Na^+^ and the influx of Ca^2+^ ions when intracellular Na^+^ concentrations ([Na^+^]i) increase or membrane depolarization occurs. In the current study, we transfected human DASMCs with NCX1-cDNA and detected an obvious reduction in cytosolic Ca2+ levels. Therefore, our results indicate that the forward mode of NCX1 is probably activated following NCX1-cDNA up-regulation in human DASMCs. This finding was further confirmed when KB-R7943 was applied to inhibit the reverse mode of NCX1 on human DASMCs. This supplementation resulted in a reduction in cytosolic Ca2+ in DASMCs. Therefore, this study reveals for the first time that NCX1 probably mediates a reduction in cytosolic Ca2+ in human DASMCs through the forward mode.

Closure of the DA after birth occurs in two steps. First, DA functional closure occurs in response to alterations in O_2_ tension following an intrinsic O_2_-sensing mechanism that occurs in DASMCs^16^. Second, the DA anatomically closes as a result of cell proliferation and migration within the intimal cushion during late gestation^17^. Many studies have indicated that Na^+^/Ca^2+^ exchanger expression can regulate the migration and proliferation of contractile cells. To further understand the role of NCX1 in the human DASMC anatomical closure process, NCX1 expression was up-regulated following transfection with NCX1-cDNA. The results of this over-expression suggested that transfected DASMCs exhibit weaker proliferation and migration capacities. This phenomenon indicated that NCX1 might mediate DA patency by delaying the anatomical closure process.

Another observation pertaining to the present study is that cytosolic Ca^2+^ is positively correlated with proliferation and migration capacities of human DASMCs. Once cytosolic Ca^2+^ was down-regulated through the inhibition of the NCX1 reverse mode, the proliferation and migration capacities of DASMCs were inhibited accordingly. Cytosolic Ca^2+^ as a critical secondary messenger that links external stimuli to cell proliferation and migration had been studied in great detail^18^. Hegyi, P *et al*. demonstrated that NCX regulates the migration and proliferation of human gastric myofibroblasts^19^. Previous studies confirmed that a decrease in cytosolic Ca^2+^ not only inhibits functional characteristics in the cytosol but it is also involved in the activation of Ca^2+^-sensitive events in the nucleus (e.g. expression of nuclear proteins related to the cell cycle)^20–23^. NCX has also recently been reported to be involved in endothelial angiogenesis^24^. Therefore, we speculate that NCX1 prevents the functional closure process and initially maintains DA patency through Ca^2+^ efflux; this in turn is likely to inhibit anatomical closure.

In summary, NCX1 is more highly expressed in fetal mouse DA as compared to the newborn, which may contribute to the patency of fetal DA, 2) Overexpression of this NCX1 in constricted human DASMC leads to decreased cytosolic Ca2+ levels, which may contribute to functional closure of DA, 3) Decreased cytosolic Ca2+ levels appear to lead to decreased cell proliferation and migration when using a drug that decreases cytosolic Ca2+ even further by inhibiting the reverse mode of this transporter, which may contribute to the anatomic closure of DA. However whether and how NCX1 is able to be manipulated to keep DA patency await for more fundamental research.

## Materials and Methods

### Ethics Statement

All procedures were approved by the Animal Welfare and Human Studies Committee at the Shanghai Jiao Tong University School of Medicine. The study conforms to the principles outlined in the Declaration of Helsinki. Guardians of participants provided their written consent to allow the children to partake in this study, which was approved by the ethics committee of Shanghai Children’s Medical Center at the Shanghai Jiao Tong University School of Medicine. Written informed consent was obtained from all subjects.

### Cell culture

Human ductus arteriosus smooth muscle cells (DASMCs) were prepared from the human infant ductus arteriosus as previously described^8^. The cells were subsequently maintained in DMEM/F12 supplemented with 10% fetal bovine serum (Life Technologies, Gaithersburg, Maryland, USA), 100 μg/mL streptomycin, and 100 U/mL penicillin at 37 °C (in a 95% air/5% CO_2_ atmosphere mix). DASMCs that had not been passaged more than five times were used in all of the experiments.

### Cell transfection

NCX1-cDNA and negative control lentiviral vector (NC) were purchased from Genechem, (Shanghai, China). Human ductus arteriosus smooth muscle cells were transfected with NCX1-cDNA or the negative control according to the manufacturer’s instructions. Transfections were performed over 8 hours, and 72 hours after transfection, cells were harvested and subjected to qRT-PCR, Western blotting, and flow cytometry assays.

### Cell proliferation assay

Human DASMC growth and dose response curve for KB-R7943 were assayed by a colorimetric procedure using Cell Counting Kit-8 (Dojindo Laboratories, Kumamoto, Japan) and a 5–20-deoxyuridine (EdU) incorporation assay. For the CCK-8 assay, 3 × 10^4^ DASMCs per well were cultured in 96-well plates. The cells were subsequently transfected with NCX1-cDNA or NC. For the dose response curve for KB-R7943 assay, 2 × 10^4^ DASMCs per well were cultured in 96-well plates. The cells were then treated with different concentration of KB-R7943 (0, 1, 10 and 100 µmol/L). CCK-8 reagent was added to the cultures according to manufacturer’s instructions and growth was assessed after 0, 24, 48, and 72 h of culture. Each experiment was repeated three times and the growth curves were presented as means ± standard deviation. For the EdU incorporation assay, the EdU assay kit (Ribobio, Guangzhou, China) was used according to the manufacturer’s instructions. A total of 3 × 10^4^ DASMCs per well were cultured in 96-well plates and then transfected with NCX1-cDNA or NC. Seventy-two hours after transfection, 100 μl of 50 mM EdU was added to each well. The cells were then fixed with 4% formaldehyde for 10 min at room temperature. They were subsequently treated with 0.5% Triton X-100 for 15 min at room temperature. After washing three times with PBS, 100 μl of 1 × Apollo reaction cocktail were added to each well and the cells were subsequently cultured at room temperature for 30 min. The cells were then stained with 100 μl of DAPI for 5 min and visualized under a fluorescence microscope (Olympus Corporation, Tokyo, Japan). The EdU-positive DASMCs were counted with ImageJ software (Media Cybernetics, Bethesda, MD, USA). Each experiment was repeated three times.

### RNA extraction and quantitative real-time PCR

Total RNA was extracted from human DA smooth muscle cells and mouse DA tissues using TRIzol reagent (Invitrogen, Carlsbad, CA, USA) according to the manufacturer’s protocol. The cDNA template was amplified by real-time (RT)-PCR using the SYBR® Premix Dimmer Eraser kit (TaKaRa, Japan). The quantitative real-time polymerase chain reaction (qRT-PCR) assay was carried out using a SYBR Premix Ex Taq II kit (TaKaRa) according to the manufacturer’s instructions. The qRT-PCR reactions were performed using an Applied Biosystems 7500 Fast Real-Time PCR System and the following reaction conditions: 1 cycle at 95 °C for 10 s, followed by 40 cycles at 95 °C for 15 s and 60 °C for 60 s. GAPDH was measured as an internal control, and messenger RNA (mRNA) values were normalized to GAPDH. The relative expression fold-change for mRNAs was calculated using the 2−ΔΔCt method. Each sample was run in triplicate. qRT-PCR results were analyzed and expressed relative to CT (threshold cycle) values, and then converted to fold-changes. The sequences of the oligonucleotide primers that were used for qRT-PCR were as follows: human NCX1 sense primer, 5′-GGGGTACATCTGGCTCTCAA-3′; NCX1 antisense primer, 5′-AACAAAATACTCCCGCGACG-3′; mouse NCX1 sense primer, 5′-CCTTGTGCATCTTAGCAATG-3′; NCX1 antisense primer, 5′-TCTCACTCATCTCCACCAGA-3′. Human GAPDH sense primer, 5′-TGCACCACCAACTGCTTAGC-3′; human GAPDH antisense primer: 5′-GGCATGGACTGTGGTCATGAG-3′. Mouse GAPDH sense primer, 5′-CGCCCTGATCTGAGGTTAAAT-3′; Mouse GAPDH antisense primer, 5′-CCACCCTAGAAAGTCCAAAGAG-3′.

### Protein extraction and Western blotting assay

Human DA smooth muscle cells and mouse DA tissues were lysed in a RIPA buffer containing a protease inhibitor cocktail tablet (Roche Diagnostics, Indianapolis, USA) and Phenylmethanesulfonyl fluoride (Sigma). Twenty micrograms of total protein were electrophoresed on a 10% SDS-PAGE gel, transferred onto a nitrocellulose blotting membrane, blocked, and then incubated overnight with antibodies against NCX1(Sigma, USA) and GAPDH (Bioworld, China). The corresponding secondary antibody was subsequently incubated with the blot at room temperature for 2 h. Immunoreactive bands were detected using an enhanced chemiluminescent substrate (Merck, Millipore Corp). The optical density of the bands was determined using a ChemiDoc Imaging System (Bio-Rad Laboratories, Hercules, CA).

### Flow cytometry analysis

A total of 5 × 10^5^ human ductus arteriosus smooth muscle cells were seeded in each well of a 6-well culture plate in an atmosphere containing 5% CO_2_ at 37 °C for 48 hours. Rhod-2/Am (Life Technologies, USA) was added until a final concentration of 5–10 µmol/L was reached. The cells were subsequently incubated for 30–60 s with 5% CO_2_ at 37 °C. Next, the cells were digested and centrifuged for 10 min at 1500 r/min. Excess dye was subsequently removed and the resultant extract was rinsed with calcium free PBS buffer. The latter washing step was repeated two times The cells were subsequently resuspended in 0.5 mL of PBS (without calcium) and analyzed using a BD FACSAria cell sorter (BD Biosciences, San Jose, CA, USA) according to the manufacturer’s protocol.

### Intracellular Calcium Imaging

A total of 4 × 105 human ductus arteriosus smooth muscle cells were seeded in each well of a 24-well confocal dedicated culture plate in an atmosphere containing 5% CO2 at 37 °C for 48 hours. Fluo3-Am (Beyotime, Shanghai, China) was added into the wells until a final concentration of 2µmol/L was reached; or Rhod2-AM (Life Technologies, USA) was added to the wells until a final concentration of 5µmol/L, according to the manufacturer’s instruction. The cells were subsequently incubated for 30 min with 5% CO2 at 37 °C. Next, Excess dye was subsequently removed and the resultant extract was rinsed with calcium free PBS buffer. Cells were then examined by confocal microscope. The imaging system included a Leica DMI6000 microscope. Fluorescent images were recorded using a Leica TSC SP8 confocal system. Fluorescence images of rhod2 were acquired using 545-nm excitation and 590-nm emission. Fluorescence images of fluo3 were acquired using 485-nm excitation and 520-nm emission. For confocal microscopy, we used standard fluorescein and rhodamine filter sets. Images were acquired in every 2 s for 3 min. For comparison, both groups of calcium images were showed at their peak time.

### Transwell assay

A 24-well Boyden chamber with a polycarbonate membrane containing 8 μm pores (Corning, USA) was used to analyze cell migration. A total of 5 × 10^5^ human DASMCs, which had been transfected with NCX1-cDNA for 72 hours, were seeded in the upper chamber with serum-free medium. Medium containing 10% serum was added to the lower chamber as a chemoattractant. The membranes were fixed at 48 h and stained with 0.4% Giemsa stain (Sigma, USA). Following the removal of non-motile cells from the top of the membranes (using cotton swabs), three visual fields pertaining to the 40× magnification lens were randomly selected and counted for each membrane.

### Statistical Analysis

Continuous data, including cell numbers, average cytosolic Ca^2+^ fluorescence intensity, protein expression and mRNA levels were expressed as means ± standard deviation. Differences were tested using the two-tailed Student’s t-test. P-values < 0.05 were considered statistically significant. Statistical analyses were performed using SAS software version 9.2 (SAS Institute Inc., Cary, NC, USA).

## Electronic supplementary material

Supplementary information
