# Supplementary material for: The Role of Na+/Ca2+ Exchanger 1 in Maintaining Ductus Arteriosus Patency
Source: Sci Rep. 2017 Aug 29;7:9826. doi: 10.1038/s41598-017-10377-z (PMC5575298; doi:10.1038/s41598-017-10377-z)
Supplement: Supplementary file 1 — Supplementary information [file 41598_2017_10377_MOESM1_ESM.docx]

**The Role of Na^+^/Ca^2+^ Exchanger 1 in Maintaining Ductus Arteriosus Patency**

Minghui Li^1*^, Chuan Jiang^3*^, Lincai Ye^1,2,3^, Shoubao Wang^1^, Haibo Zhang^1^, Jinfen Liu^3^ & Haifa Hong^1^

^1^Department of Thoracic and Cardiovascular Surgery, Shanghai Children’s Medical Center, Shanghai Jiaotong

University School of Medicine, Shanghai, 200127, China.

^2^Institute of Pediatric Translational Medicine, Shanghai Children’s Medical Center, Shanghai Jiaotong University School of Medicine, Shanghai, 200127, China.

^3^Shanghai Pediatric Congenital Heart Disease Institute, Shanghai Children’s Medical Center, Shanghai Jiaotong University School of Medicine,Shanghai, 200127, China.

*These authors contributed equally to this work. Correspondence and requests for materials should be addressed to H.H. (email: hhfsmallboat@163.com) or J.L. (email: jinfenliu2002@163.com)

**Supplementay information**

**
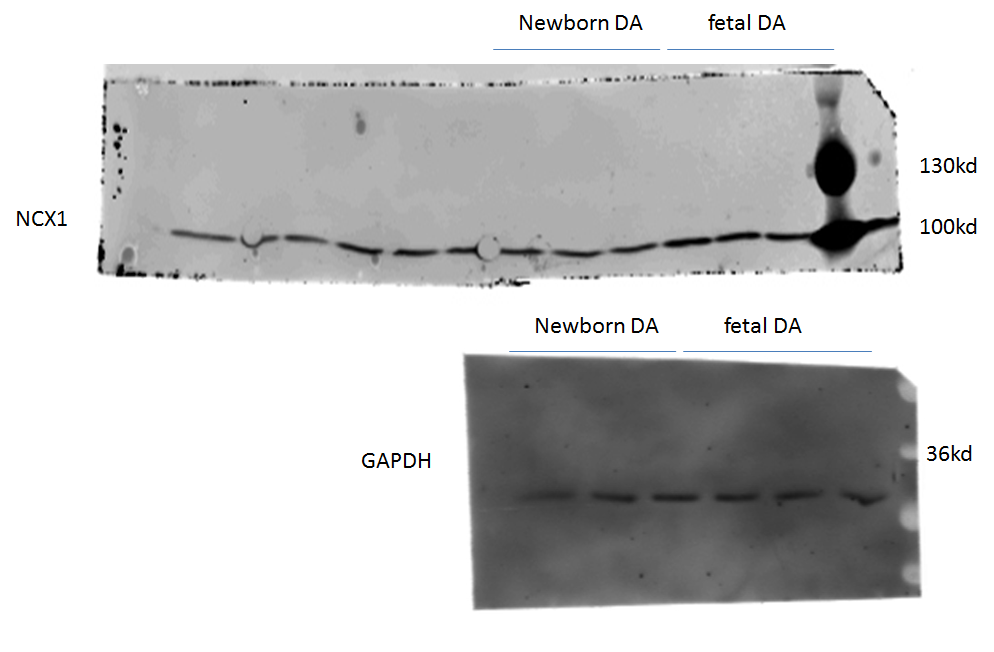
Figure-S1:** Western-blot showed that compared with newborn DA. GAPDH served as an internal control. (b) Cropped blots showed that NCX1 protein expression levels are significantly higher in fetal mouse DA compared with newborn DA. GAPDH served as an internal control.

**
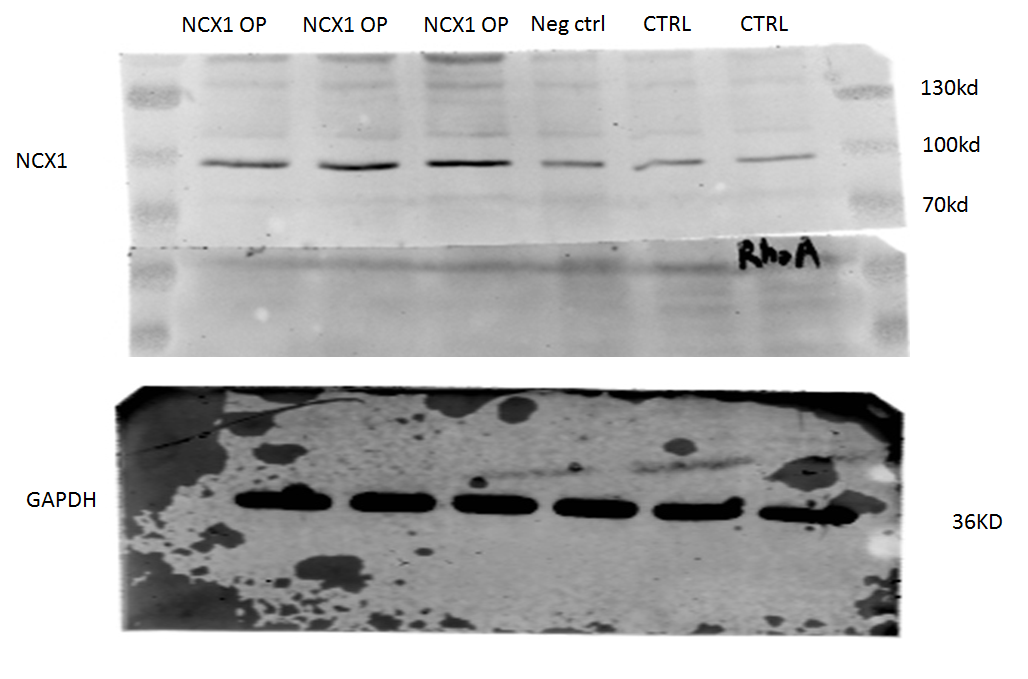
Figure-S2:** Western-blot showed that compared with the control and negative control groups, DASMCs transfected with NCX1-cDNA express higher levels of NCX1 protein.

**

**

**Figure-S3:** Dose response curve for KB-R7943 revealed that 10µm and 1µm KB-R7943 showed the similar inhibitory effect on DASMCs in vitro at 48h and 72h while 100µm KB-R7943 showed much more potent effect.
